# Hepatic Metastasis in Newly Diagnosed Esophageal Cancer: A Population-Based Study

**DOI:** 10.3389/fonc.2021.644860

**Published:** 2021-05-10

**Authors:** Huawei Li, Shengqiang Zhang, Jida Guo, Linyou Zhang

**Affiliations:** Department of Thoracic Surgery, The Second Affiliated Hospital of Harbin Medical University, Harbin, China

**Keywords:** esophageal cancer, ECHM, hepatic metastasis, esophageal cancer-specific mortality, os

## Abstract

**Background:**

The hepatic metastasis pattern of esophageal cancer (EC) has not been fully explored. The primary objective of this study was to explore the predictors of esophageal cancer with hepatic metastasis (ECHM) at the time of diagnosis. In addition, we also analyzed the factors affecting ECHM prognosis.

**Methods:**

We used the Surveillance, Epidemiology and End Result (SEER) database to identify ECHM patients at the time of initial diagnosis. The ECHM predictors were identified using multivariate logistic regression. Multivariate Cox regression and competing survival risk analyses were performed to identify factors associated with all-cause mortality and EC-specific mortality of ECHM, respectively.

**Results:**

A total of 10,965 eligible EC patients were identified in the SEER database between 2010 and 2016, of which 1,197 were ECHM patients, accounting for 10.9% of the entire cohort. In the whole cohort, eight ECHM predictors (age, primary site, grade, histology type, T staging, N staging, insurance status, and number of extrahepatic metastatic sites) were determined using multivariate logistic regression analysis. Multivariate Cox regression and multivariate competing survival risks models confirmed that the male sex, advanced age, squamous cancer, and multiple extrahepatic metastasis increased the risk of both all-cause and EC-specific mortality, whereas chemotherapy and chemotherapy plus radiotherapy significantly reduced the risk of both.

**Conclusions:**

This study explored population-level predictors of hepatic metastasis at the time of EC diagnosis and analyzed the clinical characteristics affecting the prognosis in ECHM patients. These findings may provide clinicians with a reference for the screening and treatment of hepatic metastasis in EC.

## Introduction

Esophageal cancer (EC) is the 7th most common malignancy among all cancers and the 6th leading cause of cancer-related deaths with estimated 572,000 cases and more than 508,000 deaths globally in 2018 (1, 2). The survival time in patients with advanced EC is significantly shortened, especially in those with distant metastasis (3). Moreover, nearly 40% of EC patients are diagnosed when distant metastasis has already occurred, with the 5-year survival rate in these patients being just 4% (4, 5). The dismal prognosis in these patients may be associated with the propensity of EC for metastasis, even in some cases when carcinomas are superficial; moreover, there is a lack of effective treatment for distant metastasis (6–8). Therefore, distant organ metastases of EC represent a significant cause of mortality.

According to recent reports, hepatic, lung, and bone metastases are frequently observed in EC cancer, affecting 15.6, 9.7, and 7.7% of these patients, respectively, with the liver being the most common site for distant metastasis (5, 9). Moreover, the prognosis in patients with hepatic metastasis is dismal, and it is difficult to effectively treat such cases (10). Therefore, it is particularly important to accurately predict hepatic metastases in EC at the time of diagnosis and formulate the optimal treatment plan.

The primary objective of this study was to explore the predictors of EC with hepatic metastasis (ECHM) on the population level. In addition, we aimed to analyze the factors affecting ECHM prognosis.

## Materials and Methods

### Database

We downloaded all of the data in this study from the Surveillance, Epidemiology, and End Results (SEER) database, which contains incidence data from population-based cancer registries, accounting for approximately 34.6% of the US population from 18 registration centers. SEER provides patient information up to 2016 in November 2018 and has been releasing hepatic metastasis-related information since 2010. Therefore, we were able to obtain data on ECHM patients from 2010 to 2016. In this study, we used the SEER*State (version 8.3.6) released by SEER to extract data from eligible patients.

### Study Population

**Figure 1:**
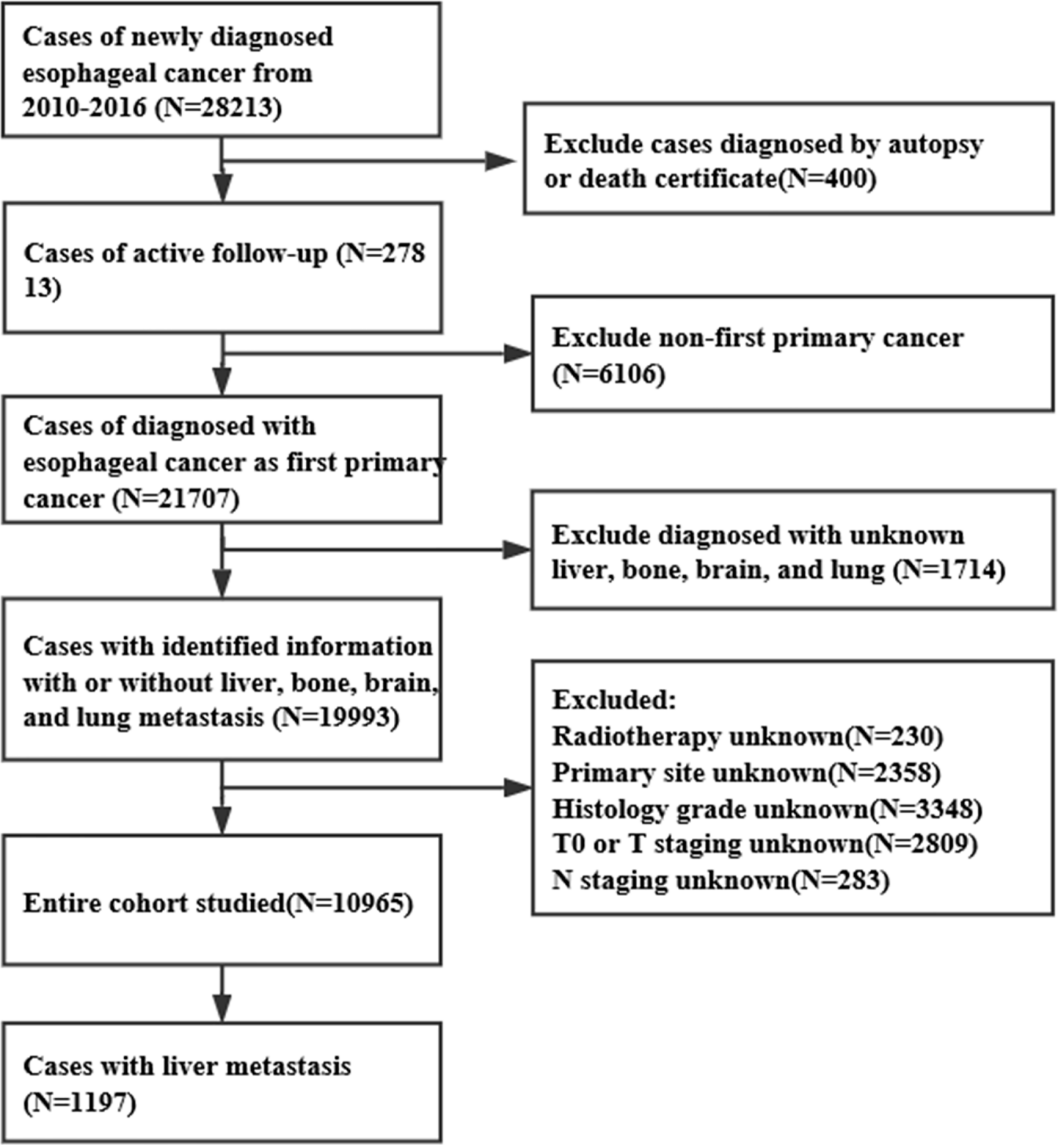
Data extraction flowchart from the SEER database.

Using the SEER database, we obtained data for 28,213 EC patients, from January 1, 2010 to December 31, 2016. The inclusion criteria were as follows: >18 years old, EC as a first primary tumor, with complete follow-up data, with complete information about hepatic metastasis, and diagnosis confirmed based on the pathology. The exclusion criteria were: ≤18 years old, without definite information about hepatic metastasis, non-first primary tumor, lack of active follow-up, and confirmed diagnosis based on autopsy or death certificate. As a result, 10,965 EC cases were included in this study, of which 1,197 were ECHM patients (**Figure 1**). To determine whether these ECHM patients had *de novo* metastasis or recurrence from previously treated locally advanced EC, we queried the IDs of all EC patients between 1975 and 2016, confirming that these ECHM patients were diagnosed with EC for the first time.

According to the SEER database, races were categorized as white, African-American, and other races. Age was classified according to three intervals (18–57, 58–77, and 78+ years old). The primary tumor site of EC included the upper third of the esophagus and cervical esophagus (upper), the middle third of the esophagus (middle), lower third of the esophagus, and abdominal esophagus (lower), and overlapping lesion of the esophagus. Histology type of EC was divided into adenocarcinoma (codes 8140–8389), squamous cell carcinoma (codes 8050–8089), and remaining codes for others. Histology grade was classified into well-differentiated (Grade I), moderately differentiated (Grade II), and poorly differentiated/undifferentiated (Grade III/IV). The TNM staging was classified according to the 7th edition of the AJCC Cancer Staging Manual of the American Joint Committee on Cancer (11). Adjuvant therapy included four categories: radiotherapy, chemotherapy, chemotherapy plus radiotherapy, and no above treatment. This study used the SEER database with no personal identifiers; therefore, the approval of an institutional review Committee or informed patient consent was not required.

### Statistical Analyses

Logistic regression analysis was performed to identify ECHM predictors. The variables with P-value <0.05 in the univariate logistic regression analysis were included in the multivariate regression. Cox regression analysis was used to determine the factors related to all-cause mortality. Then, the variables with P-value <0.05 in the univariate Cox analysis were included in the multivariate Cox regression analysis. Fine and Gray’s competing survival risk regression was used to assess EC-specific mortality (12). Survival data were estimated using the Kaplan–Meier method and the Log-rank test was used to calculate the P-value between different groups.

Statistical analyses were conducted using SPSS 23.0 (IBM Corporation, Armonk, NY, USA), except for forest plots, Kaplan–Meier survival curves, and competing risks survival analysis, which were carried out using the ‘forestplot’, ‘survival’, and ‘cmprsk’ package in R software (version 3.6.1; R Foundation), respectively. A two-sided P-value <0.05 was considered statistically significant.

## Results

### Patient Characteristics

The current study included 10,965 EC patients of which 1,197 had ECHM. The median age of the patients with ECHM was 63.0 years old, and the cohort consisted of 1,041 (86.97%) male and 156 (13.03%) female patients. Compared with older patients and those of African-American races, most ECHM patients were younger than 77 years old (n = 1,058, 88.4%) and white race (n = 1,056, 88.2%). Most of the tumors (n = 998, 83.4%) were located in the lower portion of the esophagus and the main histological type was adenocarcinoma (n = 921, 76.9%). In addition, the majority of ECHM patients were diagnosed as grade III/IV (n = 745, 62.2%) and N1 staging (n = 664, 55.5%). The clinical and socio-demographic characteristics are shown in **Table 1**.

**Table 1:** Clinical characteristics and demographic of patients with esophageal cancer with hepatic metastasis at diagnosis.

Variable	Patients, No.			Proportion of hepatic metastasis, %		Survival of ECHM patients (IQR), months
	With EC (n = 10,965)	With metastatic disease (n = 2,626)	ECHM patients (n = 1,197)	Among entire cohort	Among subset with metastatic disease	
Sex						
Female	2,098	402	156	7.4	38.8	6.0 (2.0–12.0)
Male	8,867	2,224	1041	11.7	46.8	4.0 (2.0–10.0)
Age at diagnosis (Year)						
18–57	2,526	734	346	13.7	47.1	6.0 (2.0–12.0)
58–77	6,764	1,584	712	10.5	44.9	4.0 (2.0–10.0)
78+	1,675	308	139	8.3	45.1	2.0 (1.0–6.0)
Primary site						
Upper	739	112	24	3.2	21.4	4.0 (1.0–5.0)
Middle	1,731	340	104	6.0	30.6	4.0 (2.0–9.0)
Lower	7,988	2,008	998	12.5	49.7	5.0 (2.0–10.0)
Overlapping	507	166	71	14.0	42.8	4.0 (1.0–10.0)
Grade						
I	730	69	34	4.7	49.3	6.0 (2.0–12.5)
II	4,670	917	409	8.8	44.6	6.0 (2.0–11.5)
III/IV	5,565	1,640	754	13.5	46.0	4.0 (1.0–9.0)
Histology type						
Squamous	3,087	568	181	5.9	31.9	3.0 (1.0–7.0)
Adenocarcinoma	7,050	1,826	921	13.1	50.4	5.0 (2.0–11.0)
Others^a^	828	232	95	11.5	40.9	3.0 (1.0–7.0)
T staging^b^						
T1	3,071	846	455	14.8	53.8	4.0 (1.0–10.0)
T2	1,435	188	83	5.8	44.1	6.0 (3.0–13.0)
T3	4,984	866	326	6.5	37.6	6.0 (3.0–11.0)
T4	1,475	726	333	22.6	45.9	4.0 (1.0–9.0)
N staging^b^						
N0	5,107	584	318	6.2	54.5	4.0 (1.0–10.0)
N1	4,704	1,471	664	14.1	45.1	5.0 (2.0–10.0)
N2	661	340	125	18.9	36.8	6.0 (2.0–11.0)
N3	493	231	90	18.3	39.0	4.0 (1.0–7.0)
Adjuvant therapy						
No	2,265	508	281	12.4	55.3	1.0 (0.0–2.0)
Radiotherapy	727	294	112	15.4	38.1	2.0 (1.0–4.5)
Chemotherapy	1,303	854	492	37.8	57.6	7.0 (4.0–13.0)
Radiotherapy plus Chemotherapy	6,670	970	312	4.7	32.2	7.0 (4.0–11.0)
Race						
White	9,349	2,239	1056	11.3	47.2	5.0 (2.0–10.0)
African-American	995	235	91	9.1	38.7	3.0 (1.0–7.0)
Other^c^	586	142	47	8.0	33.1	7.0 (3.0–11.0)
Unknown	35	10	3	8.6	30.0	NR
Insurance status						
Insurance	10,472	2482	1120	10.7	45.1	5.0 (2.0–10.0)
Uninsurance	335	113	62	18.5	54.9	3.0 (1.0–6.0)
Unknown	158	31	15	9.5	48.4	7.0 (1.0–12.0)
Marital status						
Married	6,171	1,472	676	11.0	45.9	5.0 (2.0–11.0)
Unmarried^d^	4,267	1,050	486	11.4	46.3	4.0 (1.0–9.0)
Unknown	527	104	35	6.6	33.7	5.0 (1.0–8.0)
Extrahepatic metastatic sites to lung, bone, brain, and others						
0	9,634	1,295	677	7.0	52.3	6.0 (2.0–12.0)
1	1,071	1,071	396	37.0	37.0	4.0 (1.0–8.0)
2	213	213	97	45.5	45.5	3.0 (1.0–7.0)
3	47	47	27	57.4	57.4	2.0 (1.0–6.0)

IQR, interquartile range; NR, not reached.

a including signet ring cell carcinoma, large cell carcinoma, small cell carcinoma, etc.

b based on the 7th edition of the AJCC.

c including American Indian/AK Native, Asian/Pacific Islander.

d including divorced, separated, single, unmarried or domestic partner, and widowed.

### Predictors for ECHM

**Figure 2:**
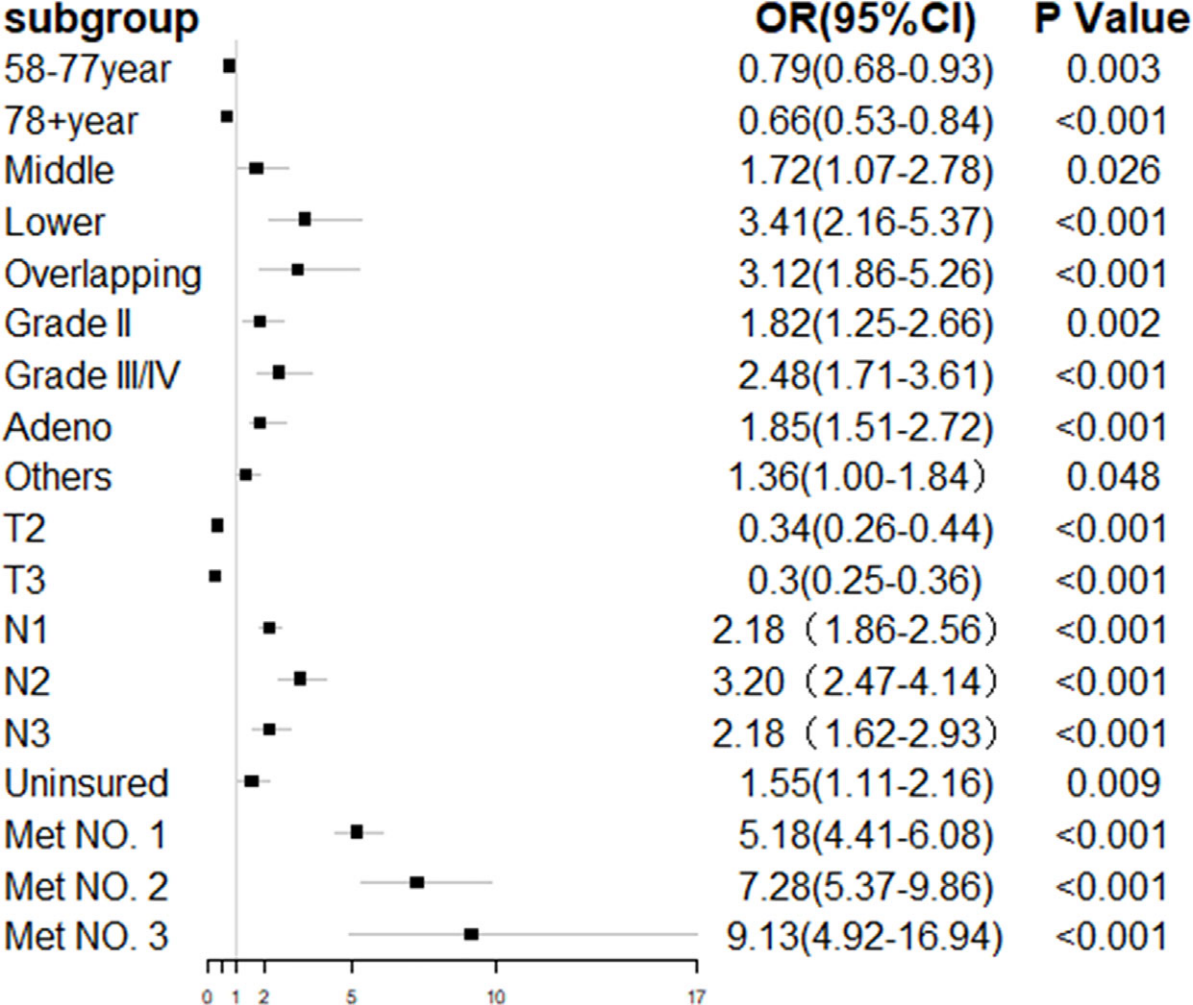
Forest plot of multivariate regression analysis for esophageal cancer with hepatic metastasis.

In the entire cohort, the univariate logistic regression analysis showed that 10 variables including gender, age, primary tumor location, tumor grade, histology type, T staging, N staging, race, insurance, marital status, and number of extrahepatic metastatic sites were significantly associated with ECHM (P < 0.05) (**Table S1**). In multivariate logistic regression analysis using these 10 variables, the age, tumor site, grade, histology type, T staging, N staging, insurance status, and number of extrahepatic metastases were independent predictors of hepatic metastasis in the entire cohort. For example, the lesion in the middle portion of the esophagus [odds ratio (OR), 1.72; P = 0.026], lower portion of the esophagus (OR, 3.41; P < 0.001), and overlapping region (OR, 3.12; P < 0.001) had a greater likelihood to be associated with hepatic metastasis than that in the upper portion of the esophagus. Compared with Grade I, Grade II (OR, 1.85; P = 0.002) and Grade III/IV (OR, 2.48; P < 0.001) had higher proportion of hepatic metastasis. The risk of hepatic metastasis in patients with N1 (OR, 2.18; P < 0.001), N2 (OR, 3.20; P < 0.001), and N3 (OR, 2.18; P < 0.001) was higher than that in patients with N0. The risk odds of hepatic metastases in uninsured patients (OR, 1.55; P = 0.009) was more than five times higher than that in insured patients. In addition, compared with no extrahepatic metastasis, one (OR, 5.18; P < 0.001), two (OR, 7.28; P < 0.001), and three extrahepatic metastases (OR, 9.13; P < 0.001) greatly increased the risk ratio of hepatic metastasis. By contrast, ages 58–77 years (OR, 0.79; P = 0.003) and >78 years (OR, 0.66; P < 0.001), and T2 (OR, 0.34; P < 0.001), and T3 (OR, 0.30; P < 0.001) were associated with lower risk of hepatic metastasis (**Table 2** and **Figure 2**). Furthermore, sex, tumor site, histology type, T staging, N staging, and number of extrahepatic metastases were related to hepatic metastasis in the subset of patients with metastatic disease (**Table 2**).

**Table 2:** Multivariate logistic regression for patients of esophageal cancer with hepatic metastasis.

Variable	Among entire cohort		Among subset with metastatic disease	
	OR (95%CI)	P-Value	OR (95%CI)	P-Value
Sex				
Male	NA	NA	1 (reference)	NA
Female	NA	NA	1.27 (1.01–1.61)	0.040
Age at diagnosis (year)				
18–57	1 (reference)	NA	NA	NA
58–77	0.79 (0.68–0.93)	0.003	NA	NA
78+	0.66 (0.53–0.84)	<0.001	NA	NA
Primary site				
Upper	1 (reference)	NA	1 (reference)	NA
Middle	1.72 (1.07–2.78)	0.026	1.43 (0.85–2.41)	0.180
Lower	3.41 (2.16–5.37)	<0.001	2.56 (1.56–4.21)	<0.001
Overlapping	3.12 (1.86–5.26)	<0.001	2.10 (1.18–3.72)	0.011
Grade				
I	1 (reference)	NA	NA	NA
II	1.82 (1.25–2.66)	0.002	NA	NA
III/IV	2.48 (1.71–3.61)	<0.001	NA	NA
Histology type				
Squamous	1 (reference)	NA	1 (reference)	NA
Adenocarcinoma	1.85 (1.51–2.72)	<0.001	1.59 (1.24–2.04)	<0.001
Others^a^	1.36 (1.00–1.84)	0.048	1.01 (0.71–1.45)	0.94
T staging^b^				
T1	1 (reference)	NA	1 (reference)	NA
T2	0.34 (0.26–0.44)	<0.001	0.67 (0.48–0.93)	0.017
T3	0.30 (0.25–0.36)	<0.001	0.55 (0.45–0.68)	<0.001
T4	1.04 (0.86–1.25)	0.698	0.83 (0.68–1.03)	0.092
N staging^b^				
N0	1 (reference)	NA	1 (reference)	NA
N1	2.18 (1.86-2.56)	<0.001	0.73 (0.59-0.89)	0.002
N2	3.20 (2.47–4.14)	<0.001	0.57 (0.42–0.76)	<0.001
N3	2.18 (1.62–2.93)	<0.001	0.60 (0.43–0.84)	0.003
Race				
White	NA	NA	1 (reference)	NA
African-American	NA	NA	1.06 (0.77–1.46)	0.713
Other^c^	NA	NA	0.74 (0.50–1.08)	0.120
Unknown	NA	NA	0.42 (0.10–1.73)	0.231
Insurance status				
Insurance	1 (reference)	NA	1 (reference)	NA
Uninsurance	1.55 (1.11–2.16)	0.009	1.48 (0.99–2.21)	0.054
Unknown	0.78 (0.43–1.41)	0.407	1.08 (0.51–2.26)	0.847
Extrahepatic metastatic sites to lung, bone, brain, and others No.				
0	1 (reference)	NA	1 (reference)	NA
1	5.18 (4.41–6.08)	<0.001	0.54 (0.45–0.64)	<0.001
2	7.28 (5.37–9.86)	<0.001	0.78 (0.57–1.05)	0.100
3	9.13 (4.92–16.94)	<0.001	1.19 (0.65–2.20)	0.570

OR, odd ratio; CI, confidence interval; NA, not applicable.

a including signet ring cell carcinoma, large cell carcinoma, small cell carcinoma, etc.

b based on the 7th edition of the AJCC.

c including American Indian/AK Native, Asian/Pacific Islander.

### Survival Assessment and Identification of Prognostic Factors in ECHM Patients

In the entire cohort, the median survival time was 11.0 months [interquartile range (IQR): 4–25 months]. By contrast, the median survival time in ECHM patients was 5.0 months (IQR: 2.0–10.0 months). ECHM significantly shortened the survival time in EC patients. However, ECHM patients who were treated with chemotherapy alone or chemotherapy plus radiotherapy had the longest survival time (7.0 months), whereas those who were not treated with adjuvant chemotherapy or radiotherapy had the shortest median survival time (2.0 months).

**Figure 3:**
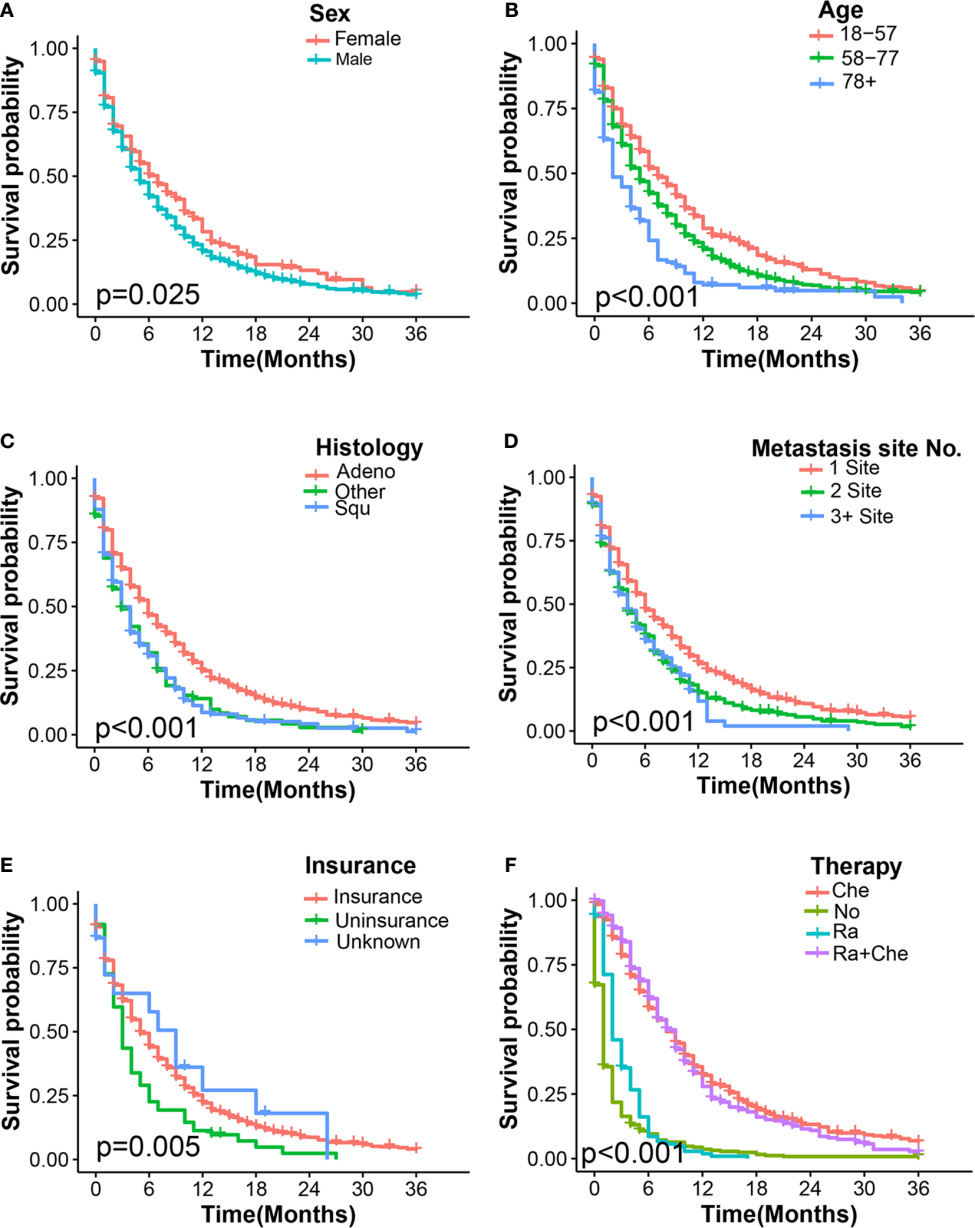
Kaplan–Meier overall survival curves of ECHM patients stratified by sex **(A)**, age **(B)**, histology type **(C)**, number of extrahepatic metastatic **(D)**, insurance **(E)**, and adjuvant therapy **(F)**.

The univariate Cox regression analysis showed that 10 clinical characteristics were related to all-cause mortality (P < 0.05) (**Table S2**). In multivariate Cox regression analysis, the male sex, advanced age, non-adenocarcinoma tissues, multiple extrahepatic metastasis, and absence of insurance were associated with the poorer overall survival in ECHM patients. For example, men [hazard ratio (HR), 1.40; P < 0.001] had a worse prognosis than women. Patients older than 78 years (HR, 1.48; P = 0.001) had a higher risk of mortality than those aged 18–57 years. In the histological type, squamous carcinoma (HR, 1.30; P < 0.003) and other histological types (HR, 1.52; P < 0.001) had a greater hazard ratio than adenocarcinoma did. Compared with patients with 0/1 extrahepatic metastases and those who had insurance, two (HR, 1.29; P < 0.001) and ≥three extrahepatic metastases (HR, 1.64; P < 0.001) and uninsured patients (HR, 1.46; P = 0.005) suffered more life-threatening consequences from EC. However, radiotherapy alone (HR, 0.70; P = 0.002), chemotherapy alone (HR, 0.25; P < 0.001), and radiotherapy plus chemotherapy (HR, 0.24; P < 0.001) were significantly related to a decreased all-cause mortality. The overall survival estimates in ECHM patients according sex, age, histology type, number of extrahepatic metastatic sites, insurance, and therapeutic schedule are shown in **Figure 3**.

In the multivariate competing survival risk analysis (**Table 3**) for EC-specific mortality among ECHM patients, the male sex (HR, 1.27; P = 0.026); squamous carcinoma (HR, 1.29; P = 0.032); and two (HR, 1.19; P = 0.025) and ≥three extrahepatic metastatic sites (HR, 1.39; P = 0.008) were associated with increased EC-specific mortality. However, the African-American race (HR, 0.68; P = 0.018), chemotherapy alone (HR, 0.40; P < 0.001), and chemotherapy plus radiotherapy (HR, 0.42; P < 0.001) were significantly associated with a decreased EC-specific mortality.

**Table 3:** Multivariate Cox regression for all-cause mortality and multivariate competing survival risk analysis for esophageal cancer-specific mortality among patients with esophageal cancer with hepatic metastasis.

Variable	All-cause mortality		Cancer-specific mortality	
	HR (95%CI)	P-Value	HR (95%CI)	P-Value
Sex				
Female	1 (reference)	NA	1 (reference)	NA
Male	1.40 (1.16–1.69)	<0.001	1.27 (1.03–1.56)	0.026
Age at diagnosis(Year)				
18–57	1 (reference)	NA	1 (reference)	NA
58–77	1.11 (0.96–1.28)	0.156	1.00 (0.87–1.16)	0.950
78+	1.48 (1.19–1.85)	0.001	1.22 (0.95–1.57)	0.100
Primary site				
Upper	NA	NA	NA	NA
Middle	NA	NA	0.70 (0.42–1.16)	0.160
Lower	NA	NA	0.65 (0.41–1.04)	0.072
Overlapping	NA	NA	0.74 (0.44–1.26)	0.260
Grade				
I	NA	NA	1 (reference)	NA
II	NA	NA	1.23 (0.77–1.94)	0.390
III/IV	NA	NA	1.29 (0.81–2.07)	0.280
Histology type				
Adenocarcinoma	1 (reference)	NA	1 (reference)	NA
Squamous	1.30 (1.10–1.55)	0.003	1.29 (1.02–1.63)	0.032
Others^a^	1.52 (1.22–1.91)	<0.001	0.99 (0.74–1.34)	0.960
T staging^b^				
T1	NA	NA	1 (reference)	NA
T2	NA	NA	0.93 (0.71–1.21)	0.580
T3	NA	NA	0.89 (0.74–1.06)	0.200
T4	NA	NA	0.96 (0.80–1.15)	0.650
N staging^b^				
N0	NA	NA	1 (reference)	NA
N1	NA	NA	1.00 (0.84–1.19)	0.990
N2	NA	NA	1.01 (0.78–1.31)	0.930
N3	NA	NA	0.74 (0.52–1.06)	0.100
Extrahepatic metastatic sites to lung, bone, brain, and others No.				
0/1	1 (reference)	NA	1 (reference)	NA
2	1.29 (1.13–1.48)	<0.001	1.19 (1.02–1.39)	0.025
3+	1.64 (1.31–2.04)	<0.001	1.39 (1.10–1.78)	0.008
Race				
White	NA	NA	1 (reference)	NA
African-American	NA	NA	0.68 (0.49–0.94)	0.018
Other^c^	NA	NA	0.89 (0.68–1.18)	0.420
Unknown	NA	NA	2.54 (0.86–7.48)	0.090
Insurance status				
Insurance	1 (reference)	NA	1 (reference)	NA
Uninsurance	1.46(1.15–1.94)	0.005	1.14 (0.82–1.59)	0.430
Unknown	0.81 (0.46–1.44)	0.589	0.98 (0.51–1.90)	0.950
Marital status				
Unmarried^d^	NA	NA	1 (reference)	NA
Married	NA	NA	1.08 (0.93–1.26)	0.290
Unknown	NA	NA	1.29 (0.84–1.90)	0.200
Adjuvant therapy				
No	1 (reference)	NA	1 (reference)	NA
Radiotherapy	0.70 (0.56–0.88)	0.002	0.78 (0.58–1.04)	0.087
Chemotherapy	0.25 (0.21–0.29)	<0.001	0.40 (0.33–0.49)	<0.001
Radiotherapy plus Chemotherapy	0.24 (0.20–0.29)	<0.001	0.42 (0.34–0.53)	<0.001

HR, Hazard Ratio; CI, confidence intervall; NA, not applicable.

a including signet ring cell carcinoma, large cell carcinoma, small cell carcinoma, etc.

b based on the 7th edition of the AJCC.

c including American Indian/AK Native, Asian/Pacific Islander.

d including divorced, separated, single, unmarried or domestic partner, and widowed.

From the results above, we can conclude that the male sex, squamous carcinoma, and multiple extrahepatic metastases not only increase the risk of all-cause mortality, but also increase the risk of EC-specific mortality. By contrast, chemotherapy and chemotherapy plus radiotherapy significantly reduce the risk of both.

In this study, we found that 6-month and 1-year survival rates in ECHM patients treated with radiotherapy alone were 16.2 and 1.9%, in those treated with chemotherapy alone were 58.1 and 32.2%, and in those treated with radiotherapy plus chemotherapy were 61.9 and 27.9%, respectively. We also found that chemotherapy or radiotherapy plus chemotherapy significantly improved median overall survival in patients with metastatic disease, especially those with ECHM (**Table 4**).

**Table 4:** Median survival time of esophageal cancer patients by extent of systemic metastatic disease.

Adjuvant therapy	Site of metastases	Survival time, Median (IQR), months	
		Extrahepatic systemic disease only	Extrahepatic systemic and hepatic metastasis
No	Brain	3.0 (1.0–5.0)	NR
	Lung	1.0 (0.0–3.0)	1.0 (0.0–2.0)
	Bone	1.0 (0.0–2.0)	1.0 (0.5–1.0)
	2 of 3	NR	0.5 (0.0–1.0)
	All 3	NR	1.0 (0.0–2.0)
	liver	NA	1.0 (0.0–3.0)
Radiotherapy	Brain	2.5 (1.0–5.0)	2.0 (1.0–NR)
	Lung	4.0 (1.5–7.0)	2.5 (1.0–5.0)
	Bone	3.0 (1.0–4.0)	3.0 (2.0–4.0)
	2 of 3	2.0 (1.0–4.0)	2.0 (1.0–2.0)
	All 3	2.0 (0.5–3.0)	2.0 (1.5–2.0)
	liver	NA	3.0 (1.0–5.0)
Chemotherapy	Brain	2.0 (2.0–NR)	NR
	Lung	9.0 (4.0–14.0)	7.0 (3.0–13.0)
	Bone	6.0 (3.0–10.0)	5.0 (3.0–10.0)
	2 of 3	4.5 (2.0–7.25)	5.0 (4.0–10.0)
	All 3	NR	NR
	liver	NA	8.0 (4.0–14.0)
Radiotherapy plus Chemotherapy	Brain	8.5 (5.0–13.0)	8.0 (4.0–10.0)
	Lung	9.0 (4.0–17.0)	7.0 (4.0–10.0)
	Bone	8.0 (4.0–14.0)	7.0 (4.0–9.0)
	2 of 3	6.0 (3.0–11.0)	6.0 (3.0–10.5)
	All 3	1.5 (1.0–2.0)	5.0 (2.0–9.0)
	liver	NA	9.0 (4.0–14.0)

IQR, interquartile range; NR, not reachedl; NA, not applicable.

## Discussion

In this research, we explored the population-level ECHM predictors. We also quantified survival assessments and examined clinical characteristics of poorer survival in ECHM patients. Previous studies have shown that the liver was the most common organ for EC distant metastasis (5, 9). In the SEER database, we found that 10.9% of EC patients had hepatic metastases at the time of diagnosis, and 45.6% of patients with metastatic disease had hepatic metastases. Hence, early identification and comprehensive therapy of hepatic metastases may change the natural course of EC and improve overall survival time and quality of life in these patients. Hence, it is of great clinical significance to explore the population-level predictors of ECHM and to provide clinicians with a resource for the assessment of hepatic metastasis risk.

Among the entire cohort, we found that lesions in the middle, lower, and overlapping parts of the esophagus, Grades II and III/IV, adenocarcinoma, and N1, N2, and N3 staging increased the risk of developing hepatic metastasis, which was similar to the previously shown risk factors of EC with distant metastasis (5, 13, 14). Moreover, older patients were less likely to suffer from hepatic metastasis than younger patients, which was also consistent with the findings of a previous study (5). We also found that cases with more extrahepatic metastatic sites and patients who had no insurance had a higher risk of developing hepatic metastasis, which had not yet been reported before, to the best of our knowledge. Furthermore, this study showed that T2 and T3 staging in the entire cohort had a lower risk for hepatic metastasis, and that T4 staging was not related to hepatic metastasis, which was similar to a previous research on EC with bone metastasis (14). We thought that this may be because the T staging was evaluated according to the depth of tumor invasion, whereas most of the T staging of advanced EC that could be resected in this study was mostly based on clinical staging; nevertheless, it was not accurate enough. In our study, N1, N2, and N3 staging might be predictors for hepatic metastasis. Previous research had also confirmed that N staging was a predictor for distant site metastasis of EC (13).

With regard to the site, tumor location in the lower and middle part of the esophagus or having overlapping lesions was associated with a higher risk of hepatic metastases than having tumors in the upper esophagus. We speculate that the majority of esophageal adenocarcinomas are located in the lower part, whereas squamous cell carcinoma often occurs in the upper region (15). Previous studies have demonstrated that circulating tumor cells could be found more commonly in the blood of patients with esophageal adenocarcinoma than in those with squamous cell carcinoma (16), suggesting that adenocarcinoma may be associated with a higher risk for distant organ metastasis. Therefore, esophageal adenocarcinoma is more prone to develop hepatic and brain metastases (9). We also found that the risk of hepatic metastasis in esophageal adenocarcinoma was 1.78 times higher than that in squamous carcinoma. Moreover, grade might be another predictor for hepatic metastasis. Grades II and III/IV were associated with significantly greater risk for hepatic metastasis than Grade I. The risk for hepatic metastasis was also greater among uninsured than insured patients, and the absence of insurance shortened overall survival time. We speculate that patients with health insurance were likely to get more early medical intervention. Nevertheless, it should be noted that in our cohort, there was a small number of patients with unknown insurance, unknown race, and unknown marital status, which may have reduced the accuracy of our results. However, these patients were not excluded from this study because the impact of these three demographic variables on hepatic metastasis was not critical to minimize the selection bias caused by the exclusion of these patients.

According to the results above, we believe that better attention should be given to patients with higher number of risk factors, such as younger patients, those with the tumor located in the middle and lower parts of the esophagus or having overlapping lesions, those with worse grade, late N staging, absence of insurance, and higher number of extrahepatic metastatic sites, who might be more susceptible to hepatic metastasis. Understanding the metastatic patterns of EC may contribute to making clinical decisions, including early diagnosis and treatment. Since the liver is the most common metastatic site, routine imaging examination as recommended by the NCCN guidelines (17) should be maintained.

The prognosis in EC patients with metastatic disease is often dismal, and the 5-year survival is only 4.8%, which is much lower than that in patients with regional and localized EC (25.1 and 46.7%, respectively) (18). Therefore, it is very important to identify the factors that affect survival in ECHM patients. Our results showed that the male sex, older age, squamous carcinoma, absence of insurance, and the presence of more metastatic sites could significantly increase all-cause mortality in ECHM patients. It is worth noting that we also used the competing risk survival model in this research, which is the most appropriate method for analyzing cancer-specific survival in the presence of other causes of death. In this model, the male sex, squamous carcinoma, and having more metastatic sites could significantly increase EC-specific mortality (**Table 3**). Moreover, to the best of our knowledge, this is the first report on population-based survival results among ECHM patients.

Recent studies demonstrated that EC patients treated with palliative chemotherapy or targeted therapy could achieve better overall survival benefits compared to those treated with the optimal supportive care alone (19). Palliative systemic chemotherapy has been considered a standard salvage treatment for ECHM patients (20). In our research, the median survival time in ECHM patients was increased by 6 months from lack of any treatment to chemotherapy or radiotherapy plus chemotherapy, suggesting that ECHM patients can benefit from chemotherapy or radiotherapy plus chemotherapy. Patients who were treated with chemotherapy or radiotherapy plus chemotherapy had the longest median survival. As EC is a highly invasive tumor, monotherapy may not yield a satisfactory outcome. At present, there are numerous types of research on multimodality treatment for hepatic metastasis. Reported treatments include stereotactic irradiation combined with systemic chemotherapy (21) systemic chemotherapy plus radiofrequency ablation and hepatectomy (22), intra-arterial chemotherapy (5-fluorouracil) (23), and chemoradiotherapy (24). However, these studies are case reports, and research with a larger sample size is necessary. In the present study, the efficacy of ECHM chemotherapy or chemotherapy plus radiotherapy was explored at the population-based level, showing that these treatments increased median survival in ECHM patients. However, further randomized clinical trials are needed.

Although our research was based on the population-level and contained a large number of cases, some limitations should not be ignored. First, important clinical information was lacking in the SEER database, including smoking status, performance status, and genetic background, among other factors. The occurrence of hepatic metastases may not be related to only the predictors we have identified in this study, but may also be affected by genetic background, smoking history, and other factors. Second, we did not have detailed information regarding metastasis, such as the size of the metastatic lesions and the exact number of metastatic lesions in the liver. Third, in the SEER database, there was a lack of detailed information about chemotherapy and radiotherapy, such as the chemotherapy drug regimen, the radiotherapy dose, the sequence of radiotherapy and chemotherapy, and the radiotherapy site. Nevertheless, a relatively homogenous group of therapeutic methods for a larger population has been determined in the database.

In this study, we found that several factors, such as the middle, lower, and overlapping lesions; worse grade; and more extrahepatic metastases, were associated with an increased risk of ECHM. Patients with these risk factors should be screened for hepatic metastasis. If EC patients are found to have hepatic metastasis and have risk factors for poor prognosis, especially among males, and those with squamous carcinoma and multiple extrahepatic metastases, we should adequately evaluate their condition and give them appropriate adjuvant therapy, such as chemotherapy or chemotherapy plus radiotherapy.

## Data Availability Statement

Publicly available datasets were analyzed in this study. This data can be found here: SEER database.

## Ethics Statement

Ethical review and approval was not required for the study on human participants in accordance with the local legislation and institutional requirements. Written informed consent for participation was not required for this study in accordance with the national legislation and the institutional requirements.

## Author Contributions

HL and LZ designed the study and analyzed the data. SZ and JG participated in data acquisition and interpretation. LZ revised the paper. All authors contributed to the article and approved the submitted version.

## Conflict of Interest

The authors declare that the research was conducted in the absence of any commercial or financial relationships that could be construed as a potential conflict of interest.
