# Supplementary material for: Hepatic Metastasis in Newly Diagnosed Esophageal Cancer: A Population-Based Study
Source: Front Oncol. 2021 May 10;11:644860. doi: 10.3389/fonc.2021.644860 (PMC8143266; doi:10.3389/fonc.2021.644860)
Supplement: Supplementary file 1 [file Table_1.doc]

**Table Supplement 1 (S1).** Univariate Logistic regression for the ECHM patients.

| **Features** | Patients, No. | | Among Entire Cohort | | Among Subset With Metastatic Disease | |
| --- | --- | --- | --- | --- | --- | --- |
|  | Patients(n=10965) | With Hepatic Metastases(n=1197) | OR(95%CI) | *P* Value | OR(95%CI) | *P* Value |
| Sex |  |  |  |  |  |  |
| Female | 2098 | 156 | 1(reference) | NA | 1(reference) | NA |
| Male | 8867 | 1041 | 1.66(1.39-1.98) | ＜0.001 | 1.39(1.12-1.72) | 0.003 |
| Age at diagnosis, Y |  |  |  |  |  |  |
| 18-57 | 2526 | 346 | 1(reference) | NA | 1(reference) | NA |
| 58-77 | 6764 | 712 | 0.74(0.65-0.85) | ＜0.001 | 0.92(0.77-1.09) | 0.325 |
| 78+ | 1675 | 139 | 0.57(0.46-0.72) | ＜0.001 | 0.92(0.71-1.21) | 0.553 |
| Primary site |  |  |  |  |  |  |
| Upper | 739 | 24 | 1(reference) | NA | 1(reference) | NA |
| Middle | 1731 | 104 | 1.90(1.21-2.99) | 0.005 | 1.62(0.97-2.68) | 0.064 |
| Lower | 7988 | 998 | 4.25(2.82-6.42) | ＜0.001 | 3.62(2.29-5.74) | ＜0.001 |
| Overlapping | 507 | 71 | 4.85(3.01-7.82) | ＜0.001 | 2.74(1.59-4.73) | ＜0.001 |
| Grade |  |  |  |  |  |  |
| I Well differentiated | 730 | 34 | 1(reference) | NA | 1(reference) | NA |
| II Moderately differentiated | 4670 | 409 | 1.97(1.37-2.81) | ＜0.001 | 0.83(0.51-1.35) | 0.452 |
| III Poorly/Undifferentiated | 5565 | 754 | 3.21(2.26-4.57) | ＜0.001 | 0.88(0.54-1.42) | 0.590 |
| Histology type |  |  |  |  |  |  |
| Squamous | 3087 | 181 | 1(reference) | NA | 1(reference) | NA |
| Adenocarcinoma | 7050 | 921 | 2.41(2.05-2.85) | ＜0.001 | 2.18(1.78-2.66) | ＜0.001 |
| Others ^a^ | 828 | 46 | 2.08(1.60-2.70) | ＜0.001 | 1.48(1.08-2.03) | 0.014 |
| T staging ^c^ |  |  |  |  |  |  |
| T1 | 3071 | 455 | 1(reference) | NA | 1(reference) | NA |
| T2 | 1435 | 83 | 0.35(0.28-0.45) | ＜0.001 | 0.68(0.49-0.93) | 0.017 |
| T3 | 4984 | 326 | 0.40(0.35-0.47) | ＜0.001 | 0.52(0.43-0.63) | ＜0.001 |
| T4 | 1475 | 333 | 1.68(1.43-1.96) | ＜0.001 | 0.73(0.60-0.89) | 0.002 |
| N staging ^c^ |  |  |  |  |  |  |
| N0 | 5107 | 318 | 1(reference) | NA | 1(reference) | NA |
| N1 | 4704 | 664 | 2.48(2.15-2.85) | ＜0.001 | 0.69(0.57-0.83) | ＜0.001 |
| N2 | 661 | 125 | 3.51(2.80-4.40) | ＜0.001 | 0.49(0.37-0.64) | ＜0.001 |
| N3 | 493 | 90 | 3.36(2.61-4.34) | ＜0.001 | 0.53(0.39-0.73) | ＜0.001 |
| Race |  |  |  |  |  |  |
| White | 9394 | 1056 | 1(reference) | NA | 1(reference) | NA |
| Black | 995 | 91 | 0.79(0.63-0.99) | 0.040 | 0.71(0.54-0.93) | 0.014 |
| Other ^b^ | 586 | 47 | 0.69(0.51-0.93) | 0.015 | 0.55(0.39-0.79) | 0.001 |
| Unknown | 35 | 3 | 0.74(0.23-2.41) | 0.613 | 0.48(0.12-1.86) | 0.289 |
| Insurance status |  |  |  |  |  |  |
| Insurance | 10472 | 1120 | 1(reference) | NA | 1(reference) | NA |
| Uninsurance | 335 | 62 | 1.90(1.43-2.52) | 0.002 | 1.49(1.01-2.16) | 0.043 |
| Unknown | 158 | 15 | 0.88(0.51-1.50) | 0.758 | 1.14(0.56-2.32) | 0.717 |
| Marital status |  |  |  |  |  |  |
| Married | 6171 | 676 | 1(reference) | NA | 1(reference) | NA |
| Unmarried | 4267 | 486 | 1.05(0.92-1.18) | 0.49 | 1.02(0.87-1.19) | 0.857 |
| Unknown | 527 | 35 | 0.58(0.41-0.82) | 0.002 | 0.60(0.39-0.91) | 0.016 |
| Extrahepatic metastatic sites to lung, bone, brain, and others No. | | | |  |  |  |
| 0 | 9634 | 677 | 1(reference) | NA | 1(reference) | NA |
| 1 | 1071 | 396 | 7.76(6.70-8.99) | ＜0.001 | 0.54(0.45-0.63) | ＜0.001 |
| 2 | 213 | 97 | 11.63(8.36-14.65) | ＜0.001 | 0.76(0.57-1.02) | 0.069 |
| 3 | 47 | 27 | 17.86(9.97-32.01) | ＜0.001 | 1.23(0.68-2.22) | 0.486 |

Abbreviations: OR, odd ratio; CI, confidence interval.

^a^ including signet ring cell carcinoma, large cell carcinoma, small cell carcinoma, etc.

^b^ including American Indian/AK Native, Asian/Pacific Islander.

^c^ based on the 7^th^ edition of the AJCC.

Table (S2). Univariable Cox regression for all-cause mortality of ECHM patients.

| **Features** | Patients, No. | | All-cause Mortality | |
| --- | --- | --- | --- | --- |
|  | Patients  (n=10965) | With Hepatic  metastases(n=1197) | HR(95%CI) | *P* Value |
| Sex |  |  |  |  |
| Female | 2098 | 156 | 1(reference) | NA |
| Male | 8867 | 1041 | 1.24(1.03-1.49) | 0.024 |
| Age at diagnosis, Y | |  |  |  |
| 18-57 | 2526 | 346 | 1(reference) | NA |
| 58-77 | 6764 | 712 | 1.28(1.11-1.47) | 0.001 |
| 78+ | 1675 | 139 | 1.96(1.59-2.43) | ＜0.001 |
| primary site |  |  |  |  |
| Upper | 739 | 24 | 1(reference) | NA |
| Middle | 1731 | 104 | 0.58(0.37-0.92) | 0.019 |
| Lower | 7988 | 998 | 0.50(0.34-0.76) | 0.653 |
| Overlapping | 507 | 71 | 0.58(0.36-0.92) | 0.022 |
| Grade |  |  |  |  |
| I Well differentiated | 730 | 34 | 1(reference) | NA |
| II Moderately differentiated | 4670 | 409 | 0.89(0.62-1.30) | 0.985 |
| III Poorly/Undifferentiated | 5565 | 754 | 1.16(0.81-1.67) | 0.327 |
| Histology type |  |  |  |  |
| Adenocarcinoma | 7050 | 921 | 1(reference) | NA |
| Squamous | 3087 | 181 | 1.52(1.29-1.80) | ＜0.001 |
| Others ^a^ | 828 | 46 | 1.54(1.23-1.92) | ＜0.001 |
| T staging ^b^ |  |  |  |  |
| T1 | 3071 | 455 | 1(reference) | NA |
| T2 | 1435 | 83 | 0.69(0.53-0.89) | 0.005 |
| T3 | 4984 | 326 | 0.74(0.64-0.87) | ＜0.001 |
| T4 | 1475 | 333 | 0.97(0.84-1.13) | 0.698 |
| N staging ^b^ |  |  |  |  |
| N0 | 5107 | 318 | 1(reference) | NA |
| N1 | 4704 | 664 | 0.84(0.73-0.96) | 0.012 |
| N2 | 661 | 125 | 0.72(0.58-0.91) | 0.005 |
| N3 | 493 | 90 | 0.86(0.66-1.12) | 0.265 |
| Extrahepatic metastatic sites to lung, bone, brain, and others No. | | | |  |
| 0/1 | 10705 | 1073 | 1(reference) | NA |
| 2 | 213 | 97 | 1.38(1.21-1.57) | ＜0.001 |
| 3+ | 47 | 27 | 1.47(1.18-1.82) | 0.001 |
| Race |  |  |  |  |
| White | 9394 | 1056 | 1（reference） | NA |
| Black | 995 | 91 | 1.23(0.97-1.54) | 0.084 |
| Other ^c^ | 586 | 47 | 0.98（0.72-1.33） | 0.893 |
| Unknown | 35 | 3 | 1.62（0.52-5.0） | 0.406 |
| Insurance status | |  |  |  |
| Insurance | 10472 | 1120 | 1（reference） | NA |
| Uninsurance | 335 | 62 | 1.49（1.15-1.94） | 0.003 |
| Unknown | 158 | 15 | 0.86（0.48-1.51） | 0.589 |
| Marital status |  |  |  |  |
| Unmarried | 6171 | 676 | 1（reference） | NA |
| Married | 4267 | 486 | 1.24（1.10-1.41） | 0.001 |
| Unknown | 527 | 35 | 1.15（0.80-1.64） | 0.450 |
| Adjuvant therapy |  |  |  |  |
| No | 2265 | 281 | 1（reference） | NA |
| Ra | 727 | 112 | 0.75(0.60-0.94) | 0.013 |
| Che | 1303 | 492 | 0.24(0.20-0.28) | ＜0.001 |
| Ra plus Che | 6670 | 312 | 0.25(0.21-0.29) | ＜0.001 |

Abbreviations: Ra, radiotherapy; Che, chemotherapy; HR, Hazard Ratio; CI, confidence interval.

^a^ including signet ring cell carcinoma, large cell carcinoma, small cell carcinoma, etc.

^b^ based on the 7^th^ edition of the AJCC.

^c^ including American Indian/AK Native, Asian/Pacific Islander.
